# Medicare Advantage Part B Premium Givebacks and Enrollment

**DOI:** 10.1001/jamahealthforum.2025.1215

**Published:** 2025-06-06

**Authors:** Mark K. Meiselbach, Andrew Anderson, Laura J. Samuel, Kali S. Thomas

**Affiliations:** 1 Department of Health Policy and Management, Johns Hopkins Bloomberg School of Public Health, Baltimore, Maryland; 2 Johns Hopkins School of Nursing, Baltimore, Maryland

## Abstract

**Importance:**

In Medicare Advantage (MA), the private component of the Medicare program that enrolls more than half of beneficiaries, an increasing share of plans are offering Part B premium givebacks to pay for part or all of the at least $174.70 Part B monthly premium. Millions of dollars of Medicare expenditures are attributable to this benefit, yet little is known about its association with member enrollment or other plan characteristics.

**Objective:**

To document trends and expenditures in MA Part B premium givebacks and examine their association with plan enrollment.

**Design, Setting, and Participants:**

This longitudinal difference-in-differences analysis compared MA plan enrollment before and after the offer of a Part B giveback among plans that offered the giveback vs plans that did not. January MA plan enrollment and characteristics data from 2018 through 2024 were included. Data were analyzed from May 2024 to February 2025.

**Exposure:**

Adoption of a Part B giveback.

**Main Outcomes and Measures:**

Total plan enrollment.

**Results:**

A sample of 18 627 plan-years representing more than 130 million enrollee-years was included in the analysis. The percentage of MA plans offering a Part B premium giveback increased from 4.3% (93 of 2187) in 2018 to 18.7% (737 of 3940) in 2024. Plans offering Part B premium givebacks had lower median enrollment, belonged to newer, higher rated contracts, had higher cost-sharing, and had lower enrollee risk scores compared with plans that did not offer givebacks. In 2024, the 3.4 million enrollees in plans with Part B givebacks received a mean (SD) of $77 ($42), amounting to as much as approximately $261 million in total monthly expenditures across the MA program. Adoption of a Part B giveback was associated with a 33.3% (95% CI, 9.3-56.9) increase in enrollment, robust to all model specifications. There was a dose-response association between the size of the giveback and enrollment.

**Conclusions and Relevance:**

In this study, the adoption of Part B premium givebacks among MA plans was associated with a substantial increase in plan enrollment. Further research will be needed to understand the total value to enrollees of Part B givebacks, which confers hundreds of millions of dollars monthly to Medicare beneficiaries.

## Key Points

**Question:**

What is the association between the adoption of Part B premium givebacks in Medicare Advantage and plan enrollment?

**Findings:**

In this study including 18 627 plan-years representing more than 130 million enrollee-years, using a difference-in-differences empirical strategy, adoption of a Part B premium giveback was associated with a substantial increase in plan enrollment, with larger enrollment gains among plans with higher Part B giveback amounts.

**Meaning:**

Part B givebacks may represent an attractive benefit to enrollees, but their total impact to enrollee satisfaction and health is unknown.

## Teaser

This study evaluates trends and expenditures in Medicare Advantage Part B premium givebacks and their association with plan enrollment.

## Introduction

More than one-half of Medicare beneficiaries are currently enrolled in Medicare Advantage (MA), which are plans offered by private insurers that receive capitated payments to manage the care of Medicare beneficiaries.^1^ MA plans submit annual bids to the US Centers for Medicare & Medicaid Services (CMS) based on their expected costs to provide traditional Medicare-covered benefits, and these bids are compared with established benchmarks to determine the plan’s payment and premium structure. When plan bids are below the benchmarks, this results in plan rebates, which are passed on to consumers in the form of lower premiums, cost-sharing, and supplemental benefits.^2,3^ From 2018 to 2024, the share of plans offering bids below benchmarks more than doubled, generating greater financing and a higher prevalence of supplementary benefits.^3^ Growth in plan rebates is believed to be a determinant of increasing demand for MA,^4^ as prior work has found that Medicare beneficiaries are more likely to choose MA over traditional Medicare where rebates are higher and enrollees consistently cite the extra benefits of MA as a key reason for driving their selection.^5,6^ While there is growing attention and evidence focused on understanding the value of individual supplemental benefits to enrollees^2,7,8,9,10,11^ offered through plan rebates, a relatively overlooked benefit in recent literature is the Medicare Part B premium giveback (sometimes called a Part B rebate or buyback).

Even if enrolled in MA, Medicare beneficiaries pay a monthly Part B premium for medical insurance that begins at $174.70 for individuals in the lowest income bracket among beneficiaries who are not eligible for Medicaid.^12^ Part B premium givebacks are offered by some MA plans to reduce enrollees’ monthly Part B premiums, which are typically deducted from beneficiaries’ social security payments. Recent reports show that approximately 19% of plans offered a Part B premium giveback in 2024,^1^ but there is a limited understanding of how many enrollees are receiving the benefit, the types of plans offering the benefit and whether there are trade-offs in the benefits they offer, and the influence of these Part B premium givebacks on beneficiaries’ enrollment decision-making.

The relative benefits and trade-offs of Part B premium givebacks can be debated. On the one hand, Part B premium givebacks are a way for plans to pass on the savings from their CMS bids in a direct and monetary way to beneficiaries.^13^ On the other hand, givebacks may be more salient to potential enrollees than other plan design features or health-related supplementary benefits. If plans offering givebacks are less generous in other ways (eg, higher copayments, fewer health-related benefits), then enrollees could select plans that may otherwise increase their total out-of-pocket health-related costs or reduce access to health care, given possible uncertainty in health needs at the time of enrollment.

In this article, we describe trends in the frequency with which Part B givebacks are offered by MA plans and their take-up by enrollees. We then compare MA plans with Part B givebacks with MA plans without Part B givebacks to understand the extent to which these plans differ in their other attributes. Finally, using a difference-in-differences research design, we examine the association between the offer of a Part B giveback and enrollment.

## Methods

### Study Data and Population

We used 2018 to 2024 publicly available plan enrollment and benefit characteristics data from CMS.^14^ Specifically, we linked January national MA plan enrollment data for each year to plan benefits databased on contract and plan identifiers. The full sample selection is shown in eAppendix 1 in Supplement 1. This study followed the Strengthening the Reporting of Observational Studies in Epidemiology (STROBE) reporting guideline.

After linking enrollment and benefits data, we limited our sample to ensure the comparability and data quality of included plans. We first limited our sample to health maintenance organization and preferred provider organization plan types offering Part D coverage and excluded employer group waiver plans and special needs plans (SNPs) due to data completeness concerns. SNPs were excluded as dual-eligible SNPs (D-SNPs) are the largest category of SNPs and many dual-eligible Medicare beneficiaries do not pay Part B premiums.^15^ Because our empirical strategy compares changes in enrollment before and after a Part B giveback is first offered by a plan, we also excluded plans that either always offered Part B givebacks during the study period and plan-years where a plan did not offer a giveback following years in which it did. We did not require that plans be present for all years of our study period.

### Outcome and Exposure Variables

The primary outcome variable for our analyses was the total enrollment in the plan in January of a given year. To account for substantial right skew in plan-level enrollment (eAppendix 2 in Supplement 1), we log-transformed this measure in regression analyses.

Our key exposure was a binary variable indicating whether a plan offered a Part B giveback in a given year, as identified in the plan benefits data. In addition, we also examined a continuous version of this measure based on the size of the Part B giveback that plans offered. Specifically, we calculated the size of the giveback as a percentage of the Part B premium for the lowest-income non-Medicaid–eligible group in each year.^12^ We further categorized these values into 20–percentage point increments to examine the impact on enrollment across the relative sizes of the giveback.

In addition, we controlled for a series of time-varying plan characteristics. The characteristics included the plan’s total MA premium (including Part A–related, Part B–related, and Part D–related premiums), contract star rating, plan out-of-pocket maximum and Part D deductibles, and whether the plan offered coverage for dental, annual eye examinations, acupuncture, meal benefits, and fitness benefits. We also controlled for changes in plan status, including indicators for whether the plan was consolidated or expanded or contracted the service area where it was offered. In 2024, in addition to the listed model covariates, we also compared plan payments, rebates, plan type, enrollee risk scores, and contract start years as well as MA county benchmarks and measures of MA market competition in the counties where plans were offered. MA market competition measures included the median number of MA plans offered among all insurers in the counties where a given plan was offered and the median Herfindahl-Hirschman Index score, calculated as the sum of the squared insurer enrollment market share in a county. Both measures were aggregated to the plan level by taking the mean across all of the counties where a plan was offered.

### Statistical Analysis

We first described trends in Part B givebacks by calculating the percentage of plans and enrollees in the sample with a Part B giveback in January of each year. Second, we compared plans with and without Part B givebacks in 2024, describing means with SDs and medians with IQRs for continuous variables and counts with percentages for categorical and binary variables. *P* values were computed using *t* tests and Wilcoxon rank-sum tests for continuous variables, depending on their skew, and Pearson χ^2^ tests for binary and categorical variables. Throughout, statistical significance was determined by a 2-tailed *P* value less than .05. Third, we plotted geographically the share of county-level MA enrollment in a Part B giveback plan in 2024.

We then investigated the association between Part B givebacks and enrollment. We first described the median plan enrollment in Part B giveback plans in the 1 year prior and following the initial Part B giveback offer and compared it with plans without givebacks in those same years. Then, we formally investigated the association by estimating difference-in-differences regressions. We estimated the association between within-plan changes in log-transformed enrollment and Part B giveback offers with plan and year fixed effects. We examined multiple specifications, including models with and without time-varying plan characteristics, to account for any co-occurring changes to plans. In addition, we also estimated regressions with the continuous specification of the giveback as a percentage of the total value of the Part B premium for the lowest-income non-Medicaid–eligible group. Robust standard errors were clustered at the plan level.

We used 2-way fixed effects as our primary specification, as this approach is more easily able to accommodate the continuous specifications of the Part B giveback. However, given concerns with this approach when there is staggered treatment adoption,^16^ we also used the Callaway and Sant’Anna estimation method to estimate the effect of the binary Part B giveback offer.^17^ We also tested the sensitivity of our primary findings by aggregating to the contract level, where total enrollment was summed across all plans under a contract and the Part B giveback indicator represents whether any plans under the contract offered a giveback, and by disaggregation to the plan-county level, where enrollment was measured at a local level. In eAppendix 3 in Supplement 1, we provide greater detail on our regression specifications. Additionally, given that Part B giveback plans tend to be smaller than plans without givebacks, we also evaluated the robustness of our primary specification when only plans with fewer than 10 000 enrollees were included. Finally, we tested the sensitivity of our estimates to accounting for plan consolidation by aggregating enrollment preconsolidation among plans that were eventually consolidated under a single contract-plan.

The primary assumption of the difference-in-differences methodology is the parallel-trends assumption that plans with and without Part B givebacks would have followed similar trends had the giveback never been offered in the giveback group. We tested the validity of this assumption in multiple ways. First, we tested for evidence of nonparallel trends in enrollment between the treatment and comparison groups prior to treatment by estimating event study models where treatment effects were estimated for each year relative to treatment onset. Second, we also evaluated whether the Part B giveback offer was associated with any concurrent changes to other plan characteristics or supplemental benefits. If plans were changing multiple benefits in conjunction with the Part B giveback, then this might bias the estimated association between the giveback offer and changes in enrollment. Given that our primary outcome is a log-transformation of enrollment, it is possible that the parallel trends assumption does not hold for untransformed levels of enrollment. Therefore, we also estimated event study models on untransformed enrollment to assess for evidence of nonparallel trends in enrollment prior to treatment. Statistical analyses were performed in R version 4.2.2 (The R Foundation) and Stata version 17 (StataCorp). Data were analyzed from May 2024 to February 2025.

## Results

### Descriptive Trends and Statistics

**Figure 1.**
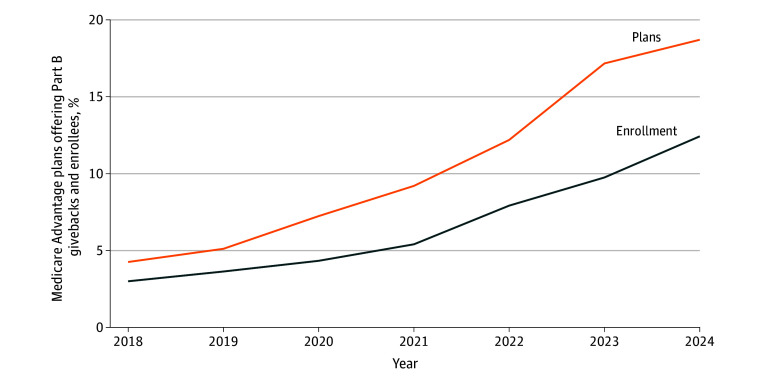
Prevalence and Enrollment in Medicare Advantage Part B Giveback Plans, 2018 to 2024 Plan enrollment represents the number of enrollees in plans offering Part B givebacks in January of the calendar year. Data are from the US Centers for Medicare & Medicaid Services Medicare Advantage public use data from 2018 through 2024.

Figure 1 shows trends in the percentage of plans offering Part B premium givebacks and the percentage of MA enrollees in those plans from 2018 to 2024. The percentage of MA plans offering Part B premium givebacks increased from 4.3% (93 of 2187) in 2018 to 18.7% (737 of 3940) in 2024. The percentage of MA enrollment in plans offering a giveback increased from 3.0% (478 361 of 15 958 252) in 2018 to 12.4% (3 409 390 of 27 440 950) in 2024. The 3.4 million enrollees in plans with Part B givebacks received a mean (SD) of $77 ($42) in 2024 amounting to as much as approximately $261 million in monthly plan expenditures, compared to approximately $20 million in monthly expenditures in 2018 (eAppendix 4 in Supplement 1).

Table 1 compares the characteristics of plans with and without Part B givebacks in 2024. In 2024, the median (IQR) Part B giveback was $75 ($50-$105) per month. In addition to offering a Part B giveback, a greater share of Part B givebacks plans had $0 total MA premiums compared with plans without Part B givebacks and also had lower plan payments, higher plan rebates, and lower enrollee risk scores. Enrollment in Part B giveback plans was smaller than in plans without givebacks (median [IQR] enrollment, 1071 [342-3462] enrollees vs 2229 [737-6833] enrollees). Plans with Part B givebacks also were associated with MA contracts that were newer and had a greater percentage of 5-star quality ratings. Part B giveback plans also had higher mean out-of-pocket maximums and Part D deductibles but similar supplemental benefits compared with plans without givebacks, and Part B giveback plans tended to be offered in counties with slightly greater competition in the MA insurance market and slightly lower MA benchmarks.

**Table 1.** Comparison of Plan Characteristics Between Medicare Advantage (MA) Plans With and Without Part B Givebacks, 2024^a^

Characteristic	Plan type, No. (%)		*P* value^b^
	No Part B giveback	Part B giveback	
Plans, No.	3495	779	NA
Plan and contract characteristics			
Part B giveback monthly amount, median (IQR), $	0 (0-0)	75.00 (50.00-105.00)	<.001
$0 Total MA monthly premium	2134 (61.1)	769 (98.7)	<.001
Plan payment, mean (SD), $	877.502 (86.968)	789.329 (115.566)	<.001
Plan rebate, mean (SD), $	168.084 (64.059)	214.093 (80.490)	<.001
Plan enrollment, median (IQR)	2229 (737-6833)	1071 (342-3462)	<.001
Plan enrollee risk score, mean (SD)^e^	1.02 (0.19)	0.89 (0.17)	<.001
Contract start year^c^			
<2006	1485 (42.5)	351 (45.1)	<.001
2006-2013	861 (24.6)	158 (20.3)	
2014-2020	803 (23.0)	162 (20.8)	
2021-2024	346 (9.9)	108 (13.9)	
Contract star rating^c^			
2	115 (3.3)	25 (3.2)	<.001
3	1171 (33.5)	206 (26.4)	
4	1824 (52.2)	410 (52.6)	
5	159 (4.5)	83 (10.7)	
Not enough data available/plan too new	226 (6.5)	55 (7.1)	
Plan type			
HMO	1287 (36.8)	325 (41.7)	<.001
HMO-POS	710 (20.3)	94 (12.1)	
Local PPO	1455 (41.6)	352 (45.2)	
Regional PPO	43 (1.2)	8 (1.0)	
Plan out-of-pocket maximum, mean (SD), $	4935.9 (1763.0)	5644.4 (1799.7)	<.001
Part D deductible, mean (SD), $	83.4 (146.0)	192.8 (216.8)	<.001
Offers comprehensive dental	3179 (91.0)	718 (92.2)	.29
Offers vision coverage	3395 (97.1)	758 (97.3)	.80
Offers acupuncture	1188 (34.0)	260 (33.4)	.74
Offers meal benefit	2529 (72.4)	557 (71.5)	.63
Offers nutrition benefit	1410 (40.3)	292 (37.5)	.14
Offers fitness benefit	3423 (97.9)	767 (98.5)	.34
Market characteristics in counties where plans were offered			
Benchmark (without quality bonus), mean (SD)	1108.081 (44.969)	1096.305 (80.535)	<.001
MA plans offered, median (IQR)^d^	32.6 (23.5-43.9)	36.000 (25.4-46.7)	<.001
County MA enrollment Herfindahl-Hirschman Index score, median (IQR)^d^	0.23 (0.18-0.28)	0.22 (0.18-0.27)	.004

Abbreviations: HMO, health maintenance organization; NA, not applicable; PPO, preferred provider organization; POS, point of service.

^a^ Data are summarized at the contract-plan level. Plan enrollment is for January 2024. Data are from the US Centers for Medicare & Medicaid Services public use data from 2024.

^b^ *P* values are computed using Wilcoxon rank-sum tests for skewed continuous variables, *t* tests for continuous normal variables, and Pearson χ^2^ test for binary and categorical variables.

^c^ Contract-level start year and star ratings are repeated if multiple plans in the study sample are under the same contract.

^d^ The median number of plans and the median Herfindahl-Hirschman Index score are summarized by taking the median value of these measures across all of the counties where each plan had at least 11 enrollees.

^e^ Plan enrollee risk scores were calculated using the US Centers for Medicare & Medicaid Services hierarchical condition category risk scores for 2024 and averaged across all enrollees in the plan.

**Figure 2.**
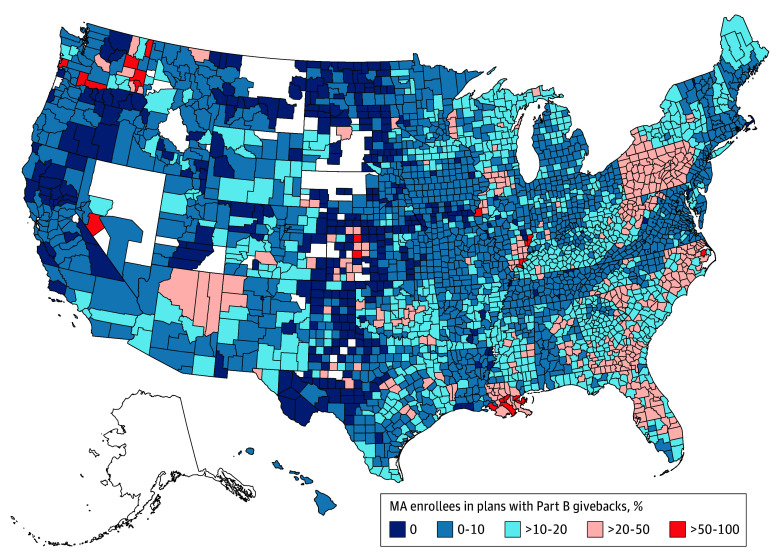
County Share of Medicare Advantage (MA) Enrollment in Plans With Part B Premium Givebacks, 2024 Plan county enrollment is for January of the calendar year. Data are from the US Centers for Medicare & Medicaid Services MA public use data from 2018 through 2024.

Figure 2 displays geographic variation in the share of MA enrollees in a plan with a Part B giveback. In 2024, there were at least 11 enrollees in a plan with a Part B giveback in 2680 counties. However, there was significant variation across counties, with 1743 counties having less than 10% of MA enrollment in Part B giveback plans and 399 counties with 20% or greater of MA enrollment in a Part B giveback plan. The share of MA enrollees in plans with Part B givebacks was consistently high across most counties in Pennsylvania and Florida.

### Association Between Part B Giveback and Enrollment

**Figure 3.**
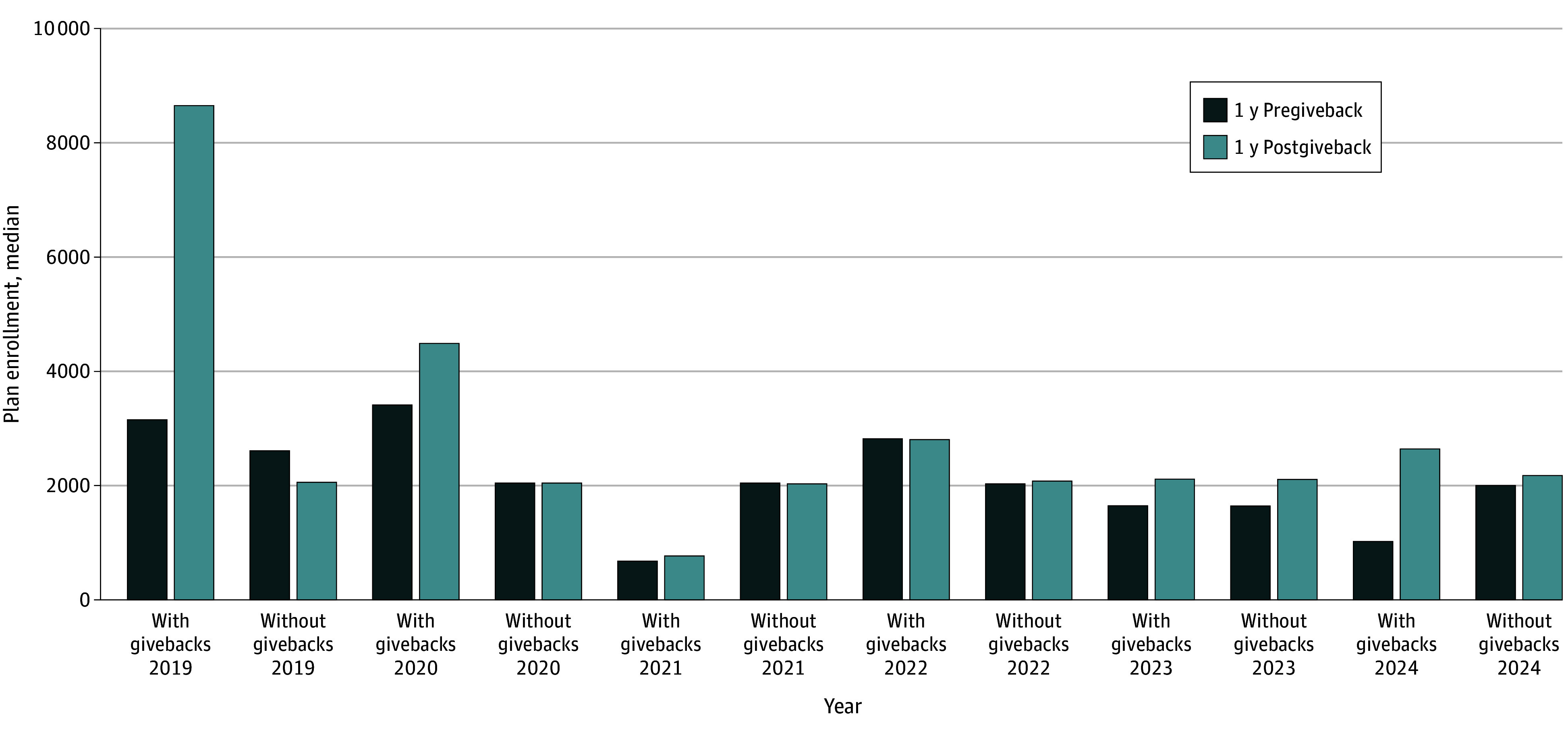
Median Plan Enrollment in the 1 Year Before and After Initial Medicare Advantage Part B Giveback Offer Among Plans With vs Without Givebacks Median plan enrollment is compared between Part B giveback plans and comparison plans without givebacks in the 1 year preceding and following a plan’s first Part B giveback offer. Plan enrollment represents the number of enrollees in a plan in January of the calendar year. Data are from the US Centers for Medicare & Medicaid Services Medicare Advantage public use data from 2018 through 2024.

Figure 3 compares median plan enrollment in the year directly preceding and following the Part B giveback offer between giveback plans, stratified by their first year they offered the benefit, and plans without givebacks in those same years. Across most years, there was a large increase in median plan enrollment among Part B giveback plans, while comparison plans experienced small changes year to year.

Table 2 estimates the association between a plan offering a Part B giveback and enrollment. Controlling for time-varying plan characteristics, the Part B giveback was associated with a 33.3% (95% CI, 9.3-56.9) increase in enrollment relative to plans that did not offer a giveback, with no evidence of differing pretrends. We found that a 1–percentage point increase in the Part B giveback as a percentage of the total Part B premium for the lowest-income non-Medicaid–eligible beneficiaries was associated with a 1.3% (95% CI, 0.2-2.5) increase in enrollment. There was a consistent dose-response association between the size of the giveback and enrollment, where the association between the Part B giveback and enrollment grew monotonically from a mean (SD) 33% (0.13) increase in enrollment for a 0% to less than 20% giveback to a 78% (0.28), 33% (0.99), 85% (0.71), and 245% (0.22) increase in enrollment for a 20% to less than 40% giveback, 40% to less than 60% giveback, 60% to less than 80% giveback, and 80% to 100% giveback, respectively (eAppendix 5 in Supplement 1).

**Table 2.** Association Between Medicare Advantage Part B Giveback Adoption and Plan Enrollment, 2018 to 2024^a^

Variable	Estimated change in enrollment, % (95% CI)	Pretrend *P* value
Any Part B premium giveback		
Without covariate adjustment	49.6 (22.4-76.7)^b^	.83
With covariate adjustment	33.3 (9.3-56.9)^b^	.20
Part B premium giveback as a percentage of the minimum Part B premium		
Without covariate adjustment	2.4 (1.4-3.5)^b^	NA
With covariate adjustment	1.3 (0.2-2.5)^c^	NA

Abbreviation: NA, not applicable.

^a^ All regressions include contract-plan and year fixed effects. Covariate adjustment included controls for the plan’s total Medicare Advantage premium (including Part A–related, Part B–related, and Part D–related premiums), contract star rating, the plan out-of-pocket maximum and Part D deductibles, and whether the plan offered coverage for dental, annual eye examinations, acupuncture, meal benefits, and fitness benefits as well as indicators for changes in plan status, such as plan consolidation or service area expansion or contraction. Data are from the US Centers for Medicare & Medicaid Services Medicare Advantage public use data from 2018 through 2024.

^b^ *P* < .01.

^c^ *P* < .05.

### Robustness Checks

We tested the robustness of the association between Part B givebacks and enrollment across various modeling specifications (eAppendix 6 in Supplement 1). The association between Part B givebacks and enrollment was robust to the Callaway and Sant’Anna method, with and without covariate adjustment, as well as specifications where the Part B giveback offer and enrollment were measured at an aggregated contract level and at a disaggregated plan-county level. Findings were also robust to including only plans with less than 10 000 maximum enrollment over the study period and to accounting for plan consolidation. Event study estimates showed no evidence of differing trends in enrollment (in log-transformed or level terms) between plans with and without Part B givebacks before the initial giveback offer (eAppendices 7 and 8 in Supplement 1). We also found no evidence that the initiation of a Part B giveback offer was associated with concurrent changes in plan characteristics or supplementary benefits (eAppendix 9 in Supplement 1).

## Discussion

This study found that the adoption of Part B premium givebacks in MA, which have rapidly increased in prevalence since 2018, was associated with significant increases in plan enrollment. We found that plans newly offering a Part B giveback saw a 33.3% (95% CI, 9.3-56.9) increase in enrollment. In addition, we found that plans with Part B givebacks belonged to newer, higher-rated contracts and had greater enrollee cost-sharing compared with plans without givebacks.

Our findings suggest that Part B giveback is a salient plan benefit that is attractive to Medicare beneficiaries, contributing to a broader literature on the drivers of MA plan enrollment.^5,10,11,18,19,20,21^ This finding runs counter to earlier work using data from 2009 and earlier that found Part B premium givebacks to not have a substantial impact on enrollment relative to lower enrollee cost-sharing or other MA premiums.^5,18^ The explanation for these findings included that Part B givebacks were not easily observable on the Medicare.gov website and payments were directly deducted from social security checks, making the benefit less visible during plan choices and on its receipt. Our results may differ from this earlier finding for multiple reasons. First, the MA market is more competitive now than it was more than a decade ago. Insurers are increasingly offering supplemental benefits and perks, like the Part B giveback, to attract consumers—particularly in competitive markets, as observed in this study.^3^ Whereas common offerings like $0 premiums and dental and vision coverage are now often described as only the table stakes to be competitive,^22,23^ Part B givebacks may allow plans to differentiate themselves in this increasingly competitive landscape. Second, many consumers are now led to their MA plans via brokers.^6,24^ While Part B givebacks are still displayed less prominently than MA plan premiums on Medicare.gov, brokers are likely to know about the benefit.

It remains an open question as to how the Part B giveback benefit impacts Medicare beneficiaries, given the observed trade-off between this benefit and enrollee cost-sharing. On the one hand, this benefit conferred hundreds of millions of dollars to beneficiaries on a monthly basis in 2024. On the other hand, higher cost-sharing may make these plans a better choice for Medicare beneficiaries with a relatively lower demand for health care services, as evidenced by their low plan enrollee risk scores. Further, recent experimental evidence is mixed as to whether an increased availability of unrestricted cash alone can improve health outcomes for all older adults.^25,26^ As rebates can be used to finance lower cost-sharing, Part B givebacks, and other plan features, it will be important to understand how trading off these plan features effects enrollee well-being, health care access, and health outcomes.

### Limitations

This study has limitations. It is possible that plans may begin offering a Part B giveback benefit in conjunction with other additional benefits. While our empirical strategy accounts for time-invariant differences between plans and controls for time-varying plan characteristics, there may be other time-varying differences we did not measure (eg, changes to provider networks).

## Conclusions

This study found that the adoption of Part B premium givebacks in MA was associated with a substantial increase in plan enrollment. It is important to understand if and how these givebacks might impact members’ health and health care utilization given observed differences in cost-sharing between plans with vs without giveback.

## Notes

Supplement 1. **eAppendix 1.** Study Sample Selection **eAppendix 2.** Probability Density Plot of Total Plan Enrollment Among Plans With and Without Part B Givebacks, 2018-2024 **eAppendix 3.** Difference-in-Differences Methodology **eAppendix 4.** Percentage of Plans and Enrollees With MA Part B Givebacks and Average and Total Part B Giveback Monthly Expenditures, 2018-2024 **eAppendix 5.** Heterogeneous Relationship Between Part B Giveback Adoption and Plan Enrollment by Percentage of Part B Premium Paid Back, 2018-2024 **eAppendix 6.** Relationship Between Part B Premium Giveback Adoption and Enrollment, Alternative Specifications **eAppendix 7.** Relationship Between Part B Premium Giveback and Log(Enrollment), Event Study **eAppendix 8.** Relationship Between Part B Premium Giveback and Enrollment, Event Study **eAppendix 9.** Relationship Between Part B Giveback Offer and Concurrent Changes in Plan Characteristics and Supplementary Benefits

Supplement 2. Data Sharing Statement
