# Supplementary material for: Medicare Advantage Part B Premium Givebacks and Enrollment
Source: JAMA Health Forum. 2025 Jun 6;6(6):e251215. doi: 10.1001/jamahealthforum.2025.1215 (PMC12144618; doi:10.1001/jamahealthforum.2025.1215)
Supplement: Supplement 1. — eAppendix 1. Study Sample Selection eAppendix 2. Probability Density Plot of Total Plan Enrollment Among Plans With and Without Part B Givebacks, 2018-2024 eAppendix 3. Difference-in-Differences Methodology eAppendix 4. Percentage of Plans and Enrollees With MA Part B Givebacks and Average and Total Part B Giveback Monthly Expenditures, 2018-2024 eAppendix 5. Heterogeneous Relationship Between Part B Giveback Adoption and Plan Enrollment by Percentage of Part B Premium Paid Back, 2018-2024 eAppendix 6. Relationship Between Part B Premium Giveback Adoption and Enrollment, Alternative Specifications eAppendix 7. Relationship Between Part B Premium Giveback and Log(Enrollment), Event Study eAppendix 8. Relationship Between Part B Premium Giveback and Enrollment, Event Study eAppendix 9. Relationship Between Part B Giveback Offer and Concurrent Changes in Plan Characteristics and Supplementary Benefits [file jamahealthforum-e251215-s001.pdf]

Supplemental Online Content

Meiselbach MK, Anderson A, Samuel LJ, Thomas KS. Medicare Advantage Part B premium givebacks and enrollment. *JAMA Health Forum*. 2025;6(6):e251215. doi:10.1001/jamahealthforum.2025.1215

eAppendix 1. Study Sample Selection

eAppendix 2. Probability Density Plot of Total Plan Enrollment Among Plans With and Without Part B Givebacks, 2018-2024

eAppendix 3. Difference-in-Differences Methodology

eAppendix 4. Percentage of Plans and Enrollees With MA Part B Givebacks and Average and Total Part B Giveback Monthly Expenditures, 2018-2024

eAppendix 5. Heterogeneous Relationship Between Part B Giveback Adoption and Plan Enrollment by Percentage of Part B Premium Paid Back, 2018-2024

eAppendix 6. Relationship Between Part B Premium Giveback Adoption and Enrollment, Alternative Specifications

eAppendix 7. Relationship Between Part B Premium Giveback and Log(Enrollment), Event Study

eAppendix 8. Relationship Between Part B Premium Giveback and Enrollment, Event Study

eAppendix 9. Relationship Between Part B Giveback Offer and Concurrent Changes in Plan Characteristics and Supplementary Benefits

This supplementary material has been provided by the authors to give readers additional information about their work.

© 2025 Meiselbach MK et al. *JAMA Health Forum*.

eAppendix 1. Study Sample Selection

Step	Number of included contract-plan-years	Number of included enrollees
1. All enrollment sample	42,689	224,456,951
2. Include HMO, HMO-POS, Local PPO, or Regional PPO	39,581	184,753,876
3. Exclude Employer Group Waiver Plans	29,854	214,183,545
4. Exclude Special Needs Plans	22,905	152,415,597
5. Exclude plans with <11 enrollees	22,533	152,415,597
6. Exclude plan-year observations after a plan stopped offering a Part B giveback	22,777	149,906,873
7. Exclude plans that always offered Part B giveback in study period	20,226	141,703,433
8. Exclude plans that do not offer Part D coverage	18,627	137,922,400

Notes: HMO = Health Maintenance Organization; PPO = Preferred Provider Organization; POS = Point of service. Data are from the Centers for Medicare & Medicaid Services Medicare Advantage public use data from 2018 through 2024.

© 2025 Meiselbach MK et al. *JAMA Health Forum*.

eAppendix 2. Probability Density Plot of Total Plan Enrollment Among Plans With and Without Part B Givebacks, 2018-2024

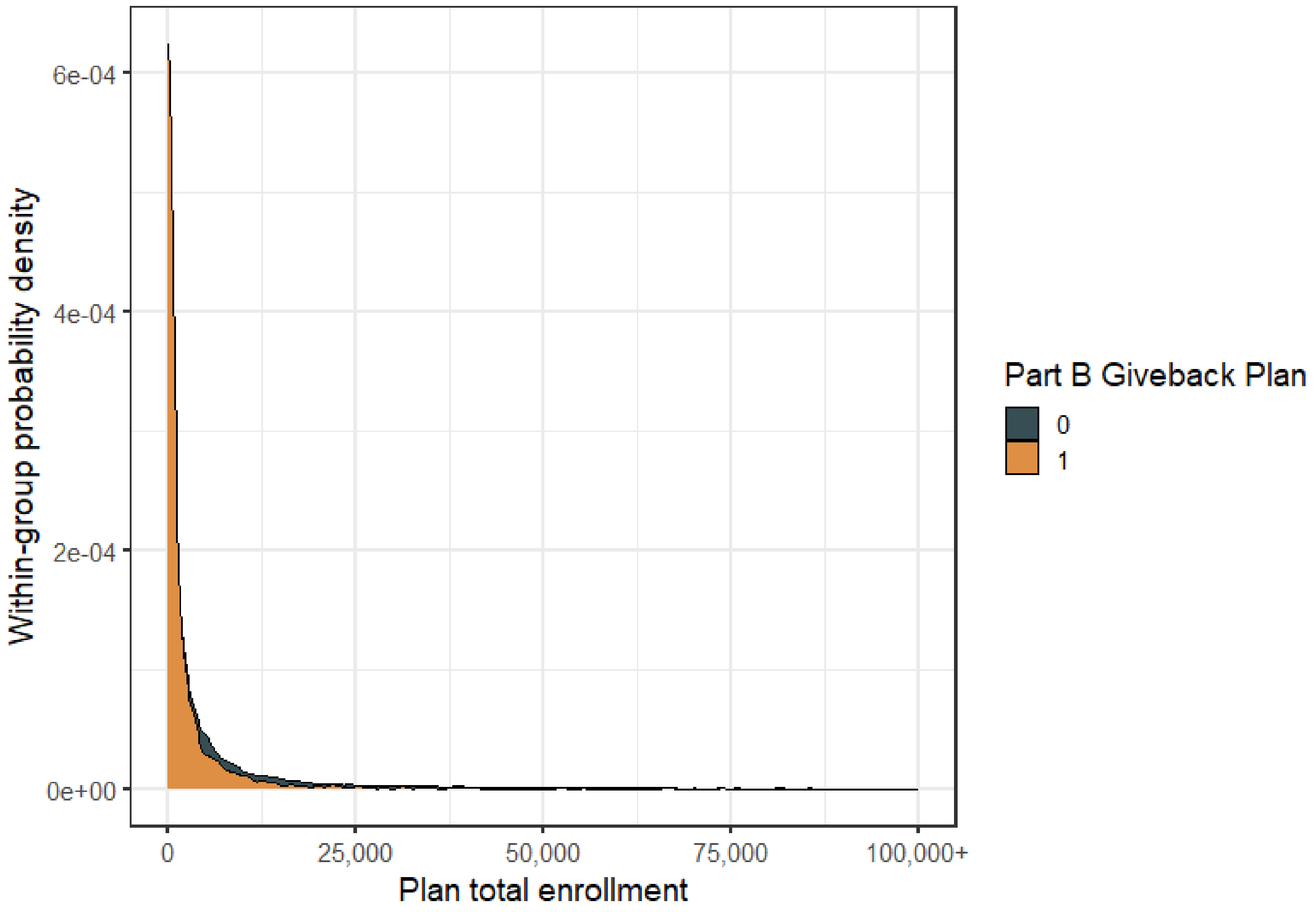

Notes: Plan enrollment is for January of the calendar year. Range of x-axis is cut off at 100,000. Data are from the Centers for Medicare & Medicaid Services Medicare Advantage public use data from 2018 through 2024.

© 2025 Meiselbach MK et al. *JAMA Health Forum*.

eAppendix 3. Difference-in-Differences Methodology

Our empirical strategy is a staggered difference-in-differences analysis that compares plans that offer Part B givebacks (treatment) to plans that do not offer Part B givebacks (comparison), before and after the offer of the Part B giveback. All regression specifications include contract-plan and year fixed effects. Therefore, our estimation strategy measures the relationship between within-plan variation in log(enrollment) over time with the Part B giveback offer. Specifically, our primary regression specification is shown below for plan *p* in contract *c* and year *t*:

$$Log(Enrollment_{pct}) = \beta_0 + \beta_1 PartBGiveback_{pct} + \beta_2 X_{pct} + \theta_{pc} + \delta_t + \epsilon_{pct} \ (1)$$

where $Log(Enrollment_{pct})$ is log-transformed contract-plan-year level enrollment. $PartBGiveback_{pct}$ is an indicator for whether a contract-plan offers a Part B giveback in a given year. In treatment plans, the indicator takes a value of 1 in the years in which the Part B giveback is offered and 0 in the years prior. In comparison plans, this indicator is always equal to 0. X_{pct} is a vector of contract and contract-plan level controls, that include the plan's total MA premium (including Part A, Part B, and Part D-related premiums), contract star rating, the plan out-of-pocket maximum and Part D deductibles, whether the plan offered coverage for dental, annual eye exams, acupuncture, meal benefits, and fitness benefits, and changes in plan "status," including indicators for whether the plan was consolidated or expanded or contracted the service area where it was offered. θ_{pc} represent contract-plan fixed effects to control for time-invariant differences in enrollment and plan characteristics across plans. δ_t represents year fixed effects to control for secular trends in enrollment and plan characteristics. ϵ_{pct} represents the error term, with robust standard errors clustered at the contract-plan level. In this regression, β_1 represents the impact of a Part B giveback offer on log(enrollment), estimated by comparing within-plan changes in log(enrollment) before vs. after the Part B giveback offer in treatment vs. comparison plans.

In addition to this primary specification, we also estimate a specification where the key independent variable is continuous, rather than binary. In this specification, the Part B giveback variable is measured as a percentage (0 to 100 percent) of the lowest standard Part B premium for non-Medicaid eligible enrollees. The regression equation is exactly the same as equation (1), except for the change to $PartBGiveback_{pct}$ to take values 0 to 100, rather than 0 or 1. In treatment plans, the variable takes a value of 0 in the years prior to the Part B offer and a non-zero value from >0 to 100 in the years the Part B giveback is offered, depending on its size. In comparison plans, this variable is always equal to 0. In this regression, β_1 represents the impact of a 1 percentage point increase in the Part B giveback offer as a percentage of the lowest standard Part B premium for non-Medicaid eligible enrollees on log(enrollment).

In addition, we test for non-linear Part B giveback effects by estimating a categorical version of the primary treatment variable, based on the continuous specification of the Part B giveback size. Specifically, we estimate the below regression equation:

$$Log\left(Enrollment_{pct}\right) = \beta_0 + \sum_{s=1}^{5} \beta_s (PartBGiveback = s)_{pct} + \beta_2 X_{pct} + \theta_{pc} + \delta_t + \epsilon_{pct} \quad (2)$$

where s takes five possible categorical indicators based on the size of the Part B giveback offer as a percentage of the lowest Part B premium for non-Medicaid eligible enrollees: >0 to <20%, 20 to <40%, 40 to <60%, 60 to <80%, and 80 to 100%. β_s represents the impact of each categorical Part B giveback size. Contract-plan-years with no Part B giveback are treated as the reference group and thus there is no corresponding β_s value.

In addition, in sensitivity analyses, we estimate regressions at the contract and contract-plan-county level, rather than the contract-plan level (as in equation (1)). Specifically, we estimate at the contract-year level:

$$Log(Enrollment_{ct}) = \beta_0 + \beta_1 PartBGiveback_{ct} + \beta_2 X_{ct} + \theta_c + \delta_t + \epsilon_{ct} \quad (3)$$

where time-varying variables are measured at the contract-year level and θ_c represents contract fixed effects. If covariates vary within a contract across plans in a given year, the variable is aggregated to the contract level by taking the maximum of binary variables (e.g., whether a plan offers dental) or the average of continuous variables (e.g., the plan premium).

At the contract-plan-county-year level, we estimate:

$$Log\left(Enrollment_{pcgt}\right) = \beta_0 + \beta_1 PartBGiveback_{pcgt} + \beta_2 X_{pcgt} + \theta_{pcg} + \delta_t + \epsilon_{pct} \quad (4)$$

where *g* represents the county in which a plan is offered. Enrollment is measured at the contract-plan-county-year level and θ_{pcg} represents contract-plan-county fixed effects.

© 2025 Meiselbach MK et al. *JAMA Health Forum*.

eAppendix 4. Percentage of Plans and Enrollees With MA Part B Givebacks and Average and Total Part B Giveback Monthly Expenditures, 2018-2024

Year	Percent of plans with Part B giveback	Percent of enrollees with Part B giveback	Total enrollees in plan with Part B giveback	Average Part B giveback among enrollees with giveback ($)	Total monthly Part B giveback expenditures ($)
2018	4.3%	3.0%	478,361	42.42	20,292,977
2019	5.1%	3.6%	645,196	50.27	32,433,540
2020	7.2%	4.3%	858,041	50.72	43,516,750
2021	9.2%	5.4%	1,178,034	54.90	64,676,057
2022	12.2%	7.9%	1,918,430	60.75	116,544,815
2023	17.2%	9.7%	2,477,854	69.06	171,125,912
2024	18.7%	12.4%	3,409,390	76.68	261,422,864

Notes: Plan enrollment is for January of the calendar year. Average Part B giveback among enrollees with giveback was calculated as a enrollment-weighted average of plan level givebacks, conditional on having a non-zero Part B giveback. Total monthly expenditures multiply this weighted average by the total number of enrollees in a plan with a Part B giveback. Data are from the Centers for Medicare & Medicaid Services Medicare Advantage public use data from 2018 through 2024.

© 2025 Meiselbach MK et al. *JAMA Health Forum*.

eAppendix 5. Heterogeneous Relationship Between Part B Giveback Adoption and Plan Enrollment by Percentage of Part B Premium Paid Back, 2018-2024

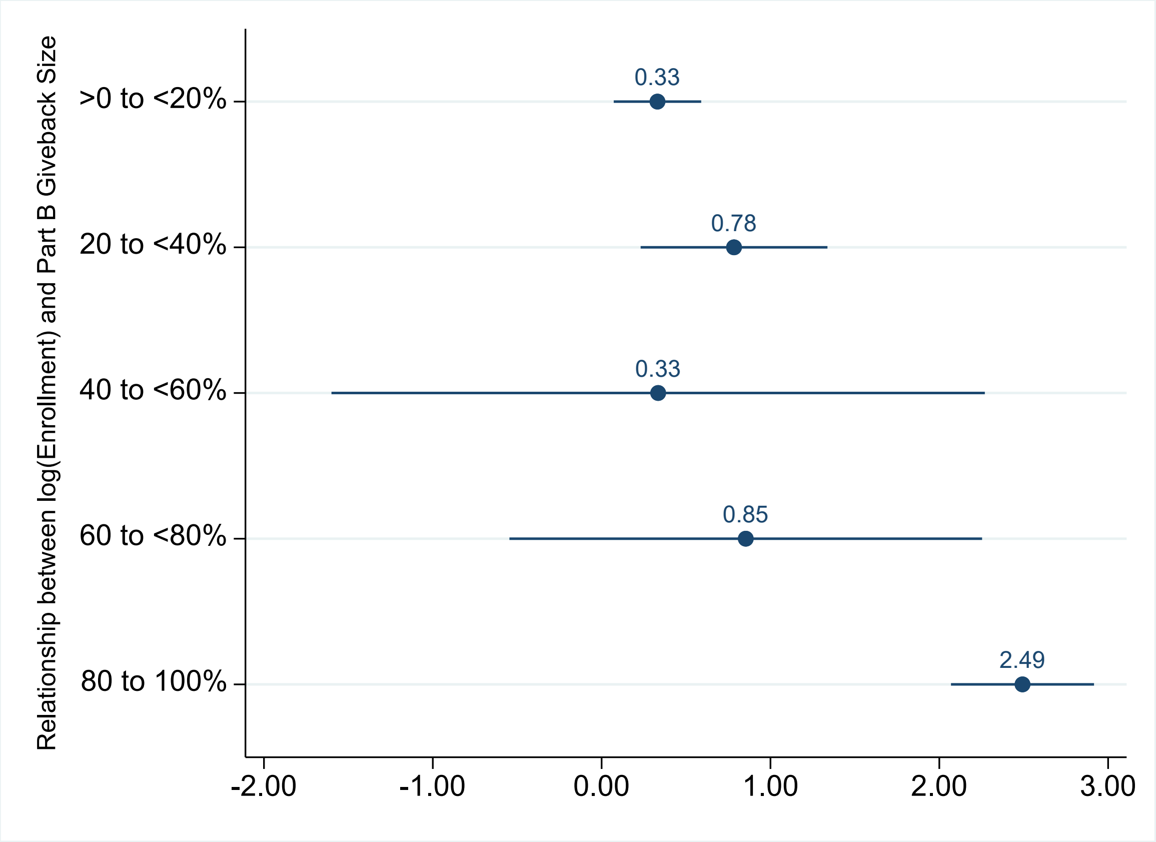

Notes: Difference-in-differences estimates of the relationship between the size of the Part B premium giveback (categorical) and log(enrollment) and 95 % confidence intervals are shown. Robust standard errors were clustered at the plan level. Part B premium giveback adoption was categorized based on the dollar amount of the giveback as a percentage of that calendar year's Part B premium for the lowest income level of non-Medicaid beneficiaries. Contract-plan and year fixed effects were included. Data are from the Centers for Medicare & Medicaid Services Medicare Advantage public use data from 2018 through 2024.

© 2025 Meiselbach MK et al. *JAMA Health Forum*.

eAppendix 6. Relationship Between Part B Premium Giveback Adoption and Enrollment, Alternative Specifications

	(1) Log(enrollment)	(2) Log(enrollmen t)	(3) Log(enrollment)	(4) Log(enrollment)	(5) Log(enrollment)	(6) Enrollment	(7) Log(enrollment)
Part B premium giveback	0.59***	0.31***	0.47***	0.26***	0.37***	744.9***	0.488***
	(0.29 - 0.89)	(0.16 - 0.46)	(0.33 - 0.62)	(0.22 - 0.30)	(0.10 - 0.64)	(199.8 - 1,290.0)	(0.23 - 0.74)
Observations	17,902	8,789	3,521	294,755	12,323	12,323	17,007
Specification	Callaway and Sant'Anna	Callaway and Sant'Anna	Contract level	Contract-plan-county level	Plans with total enrollment <10,000	Plans with total enrollment <10,000	Consolidated contracts aggregated
Plan controls	N	Y	N	N	Y	Y	Y

Notes: Difference-in-differences estimates of the relationship between Part B premium giveback adoption and log(enrollment) and 95 % confidence intervals are shown. Robust standard errors were clustered at the plan level. In all regressions, the Part B premium giveback represents the binary adoption of a giveback. Columns 1-2 implement the Callaway and Sant'Anna (2021) staggered adoption estimation method. Columns 3-6 implement two-way fixed effects, with regressions modeled at the contract (column 3), contract-plan-county (column 4), and the contract-plan (column 5-6) level. Columns 5-6 also excludes plans that ever had 10,000 or more enrollees. Covariate adjustment included controls for the plan's total MA premium (including Part A, Part B, and Part D-related premiums), contract star rating, the plan out-of-pocket maximum and Part D deductibles, whether the plan offered coverage for dental, annual eye exams, acupuncture, meal benefits, and fitness benefits, and indicators for changes in plan "status" such as plan consolidation or service area expansion or contraction. Statistical significance indicated by *** p<0.01, ** p<0.05, * p<0.1. Data are from the Centers for Medicare & Medicaid Services Medicare Advantage public use data from 2018 through 2024.

© 2025 Meiselbach MK et al. *JAMA Health Forum*.

eAppendix 7. Relationship Between Part B Premium Giveback and Log(Enrollment), Event Study

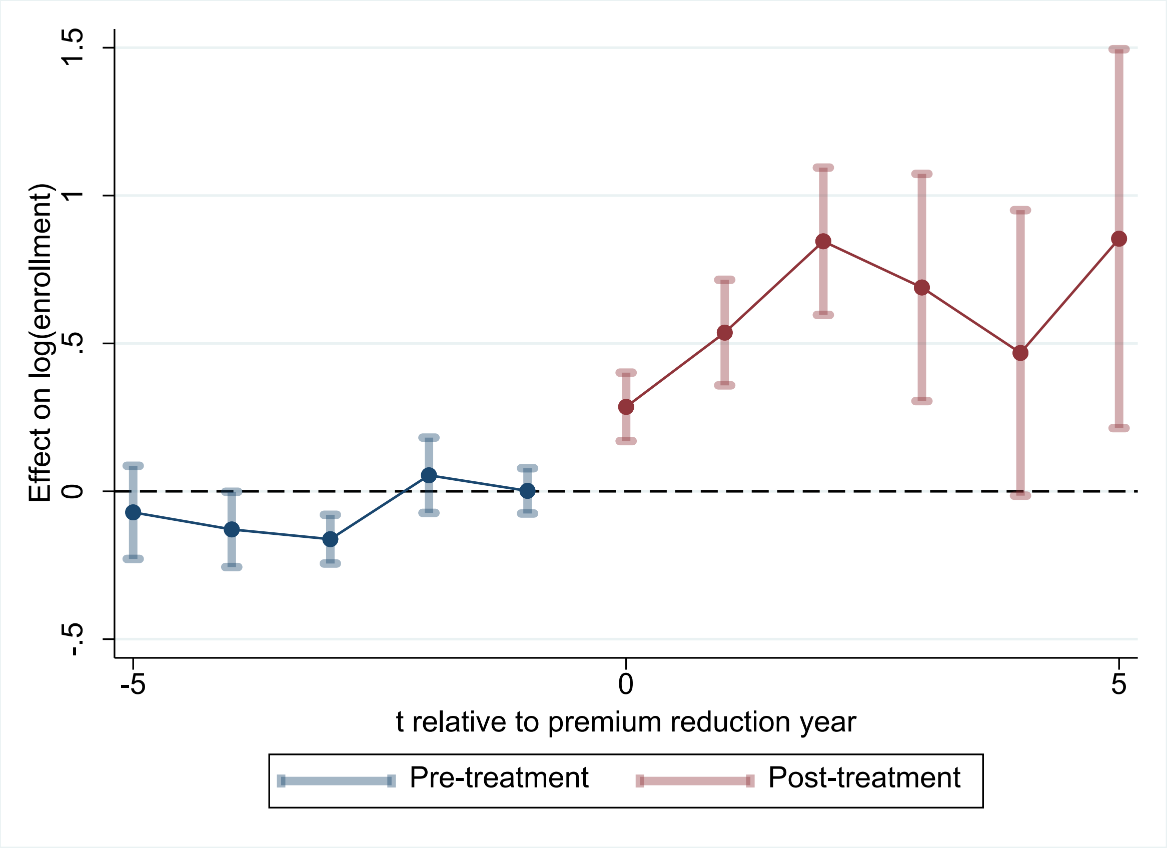

Notes: Event study estimates of the relationship between Part B premium giveback adoption and log(enrollment) and 95 % confidence intervals are shown. Event study estimates are modeled using the Callaway and Sant'Anna (2021) staggered adoption estimation method. The regression analysis included controls for the plan's total MA premium (including Part A, Part B, and Part D-related premiums), contract star rating, the plan out-of-pocket maximum and Part D deductibles, whether the plan offered coverage for dental, annual eye exams, acupuncture, meal benefits, and fitness benefits, and indicators for changes in plan "status" such as plan consolidation or service area expansion or contraction. Data are from the Centers for Medicare & Medicaid Services Medicare Advantage public use data from 2018 through 2024.

© 2025 Meiselbach MK et al. *JAMA Health Forum*.

eAppendix 8. Relationship Between Part B Premium Giveback and Enrollment, Event Study

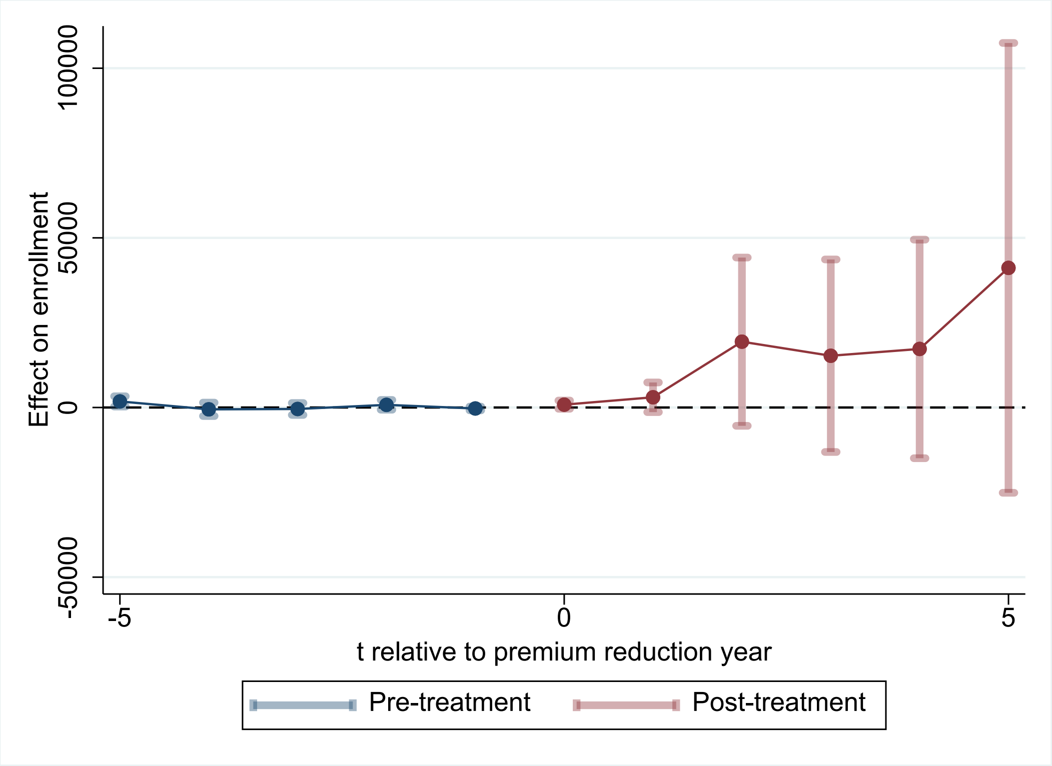

Notes: Event study estimates of the relationship between Part B premium giveback adoption and enrollment and 95 % confidence intervals are shown. Event study estimates are modeled using the Callaway and Sant'Anna (2021) staggered adoption estimation method. The regression analysis included controls for the plan's total MA premium (including Part A, Part B, and Part D-related premiums), contract star rating, the plan out-of-pocket maximum and Part D deductibles, whether the plan offered coverage for dental, annual eye exams, acupuncture, meal benefits, and fitness benefits, and indicators for changes in plan "status" such as plan consolidation or service area expansion or contraction. Data are from the Centers for Medicare & Medicaid Services Medicare Advantage public use data from 2018 through 2024.

© 2025 Meiselbach MK et al. *JAMA Health Forum*.

eAppendix 9. Relationship Between Part B Giveback Offer and Concurrent Changes in Plan Characteristics and Supplementary Benefits

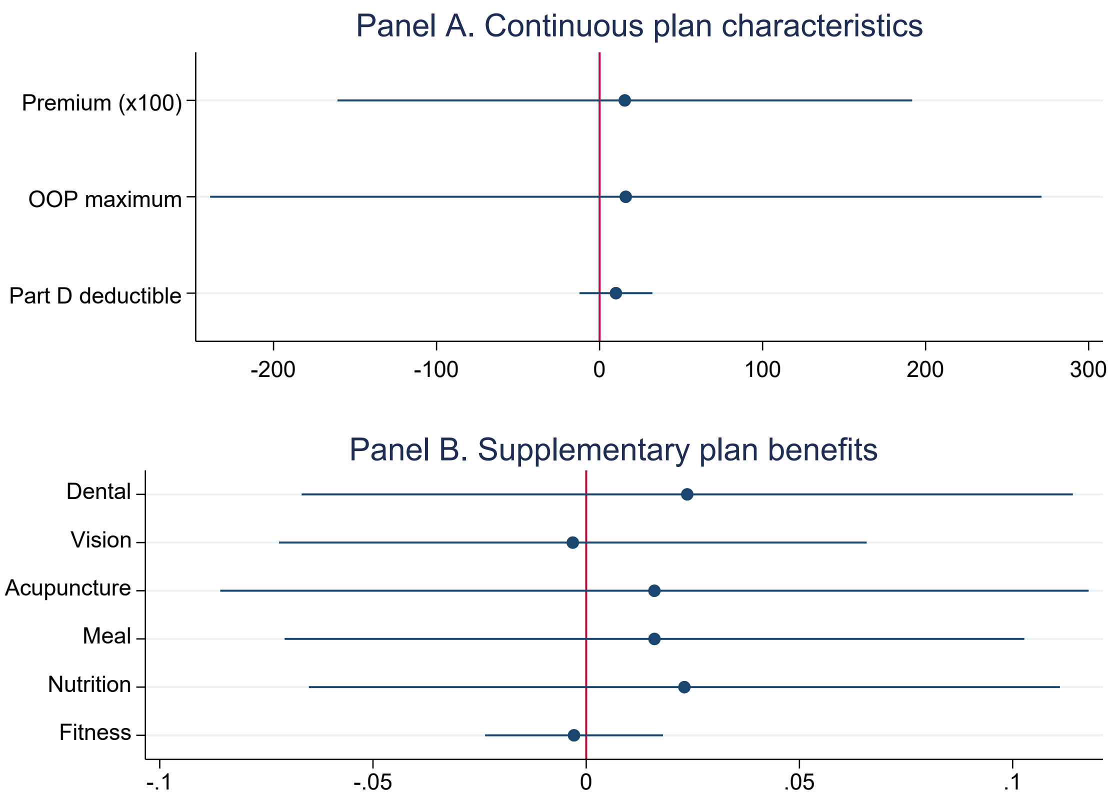

Notes: Each plan characteristics and supplementary plan benefit listed on the y-axis were treated as outcome measures in separate regressions. Each dot and line represent the coefficient and 95 % confidence interval, respectively, for the Part B giveback indicator from each of these regressions. Contract-plan and year fixed effects were included in all regressions. Robust standard errors were clustered at the plan level. The premium includes the total Part A, B, and D total premium, multiplied by 100. OOP maximum = out of pocket maximum. Data are from the Centers for Medicare & Medicaid Services Medicare Advantage public use data from 2018 through 2024.

© 2025 Meiselbach MK et al. *JAMA Health Forum*.
