# Supplementary material for: Medicare Advantage Part B Premium Givebacks and Enrollment
Source: JAMA Health Forum. 2025 Jun 6;6(6):e251215. doi: 10.1001/jamahealthforum.2025.1215 (PMC12144618; doi:10.1001/jamahealthforum.2025.1215)
Supplement: Supplement 2. — Data Sharing Statement [file jamahealthforum-e251215-s002.pdf]

Data Sharing Statement

Meiselbach. Medicare Advantage Part B Premium Givebacks and Enrollment. *JAMA Health Forum*. Published June 06, 2025. doi:10.1001/jamahealthforum.2025.1215

Data
Data available: No
